# 'Systems toxicology' approach identifies coordinated metabolic responses to copper in a terrestrial non-model invertebrate, the earthworm *Lumbricus rubellus*

**DOI:** 10.1186/1741-7007-6-25

**Published:** 2008-06-03

**Authors:** Jacob G Bundy, Jasmin K Sidhu, Faisal Rana, David J Spurgeon, Claus Svendsen, Jodie F Wren, Stephen R Stürzenbaum, A John Morgan, Peter Kille

**Affiliations:** 1 Department of Biomolecular Medicine, Division of Surgery, Oncology, Reproductive Biology, and Anaesthetics (SORA), Faculty of Medicine, Imperial College London, SW7 2AZ, London, UK; 2 Centre for Ecology and Hydrology, Monks Wood, Abbots Ripton, Huntingdon PE28 2LS, UK; 3 School of Biosciences, University of Cardiff, Main Building, Park Place, Cardiff, CF10 3TL, UK; 4 School of Biological Sciences, University of Bristol, Woodland Road, Bristol, BS8 1UG, UK; 5 School of Biomedical and Health Sciences, Pharmaceutical Sciences Division, King's College London, Franklin Wilkins Building, Stamford Street, London, SE1 9NH, UK

## Abstract

**Background:**

New methods are needed for research into non-model organisms, to monitor the effects of toxic disruption at both the molecular and functional organism level. We exposed earthworms (*Lumbricus rubellus *Hoffmeister) to sub-lethal levels of copper (10–480 mg/kg soil) for 70 days as a real-world situation, and monitored both molecular (cDNA transcript microarrays and nuclear magnetic resonance-based metabolic profiling: metabolomics) and ecological/functional endpoints (reproduction rate and weight change, which have direct relevance to population-level impacts).

**Results:**

Both of the molecular endpoints, metabolomics and transcriptomics, were highly sensitive, with clear copper-induced differences even at levels below those that caused a reduction in reproductive parameters. The microarray and metabolomic data provided evidence that the copper exposure led to a disruption of energy metabolism: transcripts of enzymes from oxidative phosphorylation were significantly over-represented, and increases in transcripts of carbohydrate metabolising enzymes (maltase-glucoamylase, mannosidase) had corresponding decreases in small-molecule metabolites (glucose, mannose). Treating both enzymes and metabolites as functional cohorts led to clear inferences about changes in energetic metabolism (carbohydrate use and oxidative phosphorylation), which would not have been possible by taking a 'biomarker' approach to data analysis.

**Conclusion:**

Multiple post-genomic techniques can be combined to provide mechanistic information about the toxic effects of chemical contaminants, even for non-model organisms with few additional mechanistic toxicological data. With 70-day no-observed-effect and lowest-observed-effect concentrations (NOEC and LOEC) of 10 and 40 mg kg^-1 ^for metabolomic and microarray profiles, copper is shown to interfere with energy metabolism in an important soil organism at an ecologically and functionally relevant level.

## Background

Understanding biological responses to individual toxic chemicals and chemical classes is clearly of key importance for pollution assessment, both for monitoring exposure to existing environmental contamination and for informing the risk assessment of off-target effects. However, ecotoxicological research frequently focuses only on easily measurable endpoints, typically mortality, although more sensitive tests on effect endpoints such as reproduction and growth are also used widely. Thus, a major challenge for ecotoxicology is understanding toxic mechanisms at a molecular level, and how these molecular changes relate to functional changes at the organism and population level [1]. The 'ecotoxicogenomic' post-genomic approach has clear benefits, and is currently generating interest from end users such as regulatory authorities as well as from research scientists [2,3]. In order for this potential to be realised, a solid bedrock of research is needed to characterise the fundamental responses of important test organisms to a range of model toxins covering a wide chemical space. It will be important to determine just how specific omic fingerprints of toxicity are, and whether they can be used successfully to distinguish between different modes of toxic action, and hence yield novel information on mechanistic toxicology. This 'systems toxicology' approach has been applied in widely used model organisms such as the laboratory rat and other vertebrates [4-7]. However, these animal models have the benefit of many more existing data [8,9]. In addition, it is often easier to perform manipulative experiments, and there is a much greater scope for complementary mechanistic cell-based work, such as histopathology. In contrast, the situation with non-model, ecologically relevant species is quite different.

The term 'ecologically relevant' is not precisely defined: clearly the most relevant level for studying the effects of chemicals is the community and/or ecosystem, and there are approaches which aim to understand, or at least quantify, responses to pollution at this level (see, for example, [10-14]). Here, however, we refer to controlled studies on single species that may already be widely studied but are not classic model organisms; for example, animals used in regulatory ecotoxicity tests fall into this category, such as the earthworm *Eisenia fetida*, the enchytraeid *Enchytraeus albidus*, and collembolans *Folsomia candida *and *Orchesella cincta *for terrestrial, and *Daphnia magna*, *Gammarus pulex*, chironomid larvae and *Mytilus *species for aquatic testing. Working with these animals presents some common challenges: none has a fully sequenced genome; it is not generally possible to obtain antibodies against specific molecular targets; they are often so small as to preclude ready dissection of internal organs or tissues; it is impossible or extremely difficult to modulate gene activity, for example by creating knockout strains; and there is in general much less knowledge about fundamental biological systems, such as signalling pathways or gene regulation, in these organisms. Modern omic approaches offer a potential opportunity to circumvent some of these drawbacks [15-22]. In particular, metabolomics and metabonomics have one great advantage for work with non-model organisms: because metabolites are detected directly, and primary metabolites at least are identical across different species, samples can trivially be analysed with no need for prior knowledge of the gene and protein sequences [23]. Metabolomics also reports on the final integrated phenotype of an organism, as metabolism is the final downstream product of gene and enzyme regulation [24-27]. As a consequence, we decided to carry out an integrative study of the metabolic response of *Lumbricus rubellus *to copper, using both nuclear magnetic resonance (NMR)-based metabolic profiling and cDNA microarrays for transcript profiling.

The earthworm *L. rubellus *is a common species with a worldwide distribution [28-31]. It is found even in shallow and contaminated soils, so it is appropriate for both laboratory and field studies [32]. It has also been the subject of an expressed sequence tag (EST) sequencing project, permitting the construction of cDNA microarrays [33]. Copper is an essential element that is also highly toxic to soil invertebrates in high concentrations. Hence, as well as inducing general toxic-response pathways, there will also be specific biological mechanisms for copper handling that may be expected to be perturbed, and thus copper is an excellent model toxin for demonstrating an integrative ecotoxicogenomics approach. We exposed worms to sub-lethal levels of copper in a semi-field situation using buried mesocosms, and monitored the dose response using transcriptomics (using a cDNA microarray fabricated with 8,129 EST reporters representative of all consensus gene objects generated from 17,225 high-quality ESTs) and metabolomics (using proton NMR spectroscopy). We also measured changes in reproduction and general condition as measured by body weight and by a well-characterised cellular bioassay; monitoring these functional endpoints is important for phenotypic anchoring of the omic data [15,34]. Our aim was to look for links between the different levels of information (metabolism, transcription and functional) to obtain stronger inferences about the mechanistic effects of copper than could be gathered from the individual datasets alone. A secondary aim was to test the hypothesis that copper exposure up-regulates histidine metabolism in *L. rubellus *[35,36].

## Results and discussion

Ecological and functional endpoints (survival, weight change, reproduction rate and neutral red retention by coelomocytes) have been reported in a previous study [37]. In brief, there was no effect on mortality, confirming that the exposure was appropriate for probing sub-lethal molecular responses, but the other functional assays all showed a response at medium to high levels of copper (40 to 160 mg/kg and above).

### Metabolomic analysis

**Figure 1:**
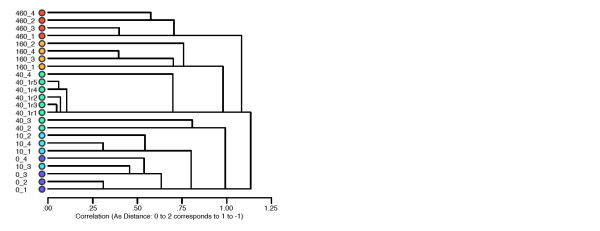
**Hierarchical cluster analysis of samples according to fitted metabolite concentrations (scaled to unit variance)**. Samples 40_1r1 to 40_1r5 represent five instrument replicates, demonstrating that neither the machine variation nor the peak fitting procedure introduce significant amounts of error compared with the within-group biological variation.

We were able to reliably profile 42 different small-molecule metabolites across all samples using ^1^H NMR spectroscopy and software for assisted manual fitting of chemical standards (Table 1). The manual fitting approach has been shown to give high-quality data on single compound concentrations [38]. The metabolomic analysis rests critically on the quality of the data obtained from this step; given the high degree of spectral overlap in one-dimensional proton spectra, and consequent difficulty in fitting the data, care is needed for reliable assignment. As well as assignment, the quality and reproducibility of the spectral fitting step is also a valid concern. We acquired data for one sample for five instrumental replicates; hierarchical cluster analysis (HCA) of all compound concentrations shows that these replicates are more similar than any other two sample spectra (Figure 1). This confirms not only, as expected, the high instrument precision of NMR [39], but also that our fitting of compounds was very reproducible. Compound assignments were made on the basis of the chemical shift and multiplicity of standards in the Chenomx software library and from online databases [40]. In addition, a two-dimensional correlated spectroscopy (COSY) spectrum was acquired for a representative sample, and used to help confirm assignments. We have assigned 2-hexyl-5-ethyl-furan-3-sulfonic acid (HEFS) previously, and lombricine is assigned on the basis of its structure and expected high concentration in earthworms [41-44]. We also ran spectra of authentic compounds and spiked them into the sample when assignments were doubtful, for example, for purine nucleotides, which have singlet resonances in the aromatic region that are close in frequency, and are thus usually unreliable to assign from database values alone. In addition, a compound which we had previously tentatively assigned as *N*-α-methylhistidine [45] was found to be *N*,*N*-α-dimethylhistidine (DMH) after comparison with authentic standards. However, we could not obtain a sufficiently pure sample of DMH to use for quantitation, so we averaged concentrations for 1-methylhistidine and dimethylglycine, fitted to the imidazole and *N*-methyl protons of DMH, respectively. We were able to fit almost all of the peaks in the spectra (Additional file 1). The few remaining unfitted resonances include some at around 8.00 and 6.00 ppm (probable pyrimidine nucleotides), 6.60 and 1.16 ppm (believed to be an uncharacterised metabolite/breakdown product of HEFS) and 5.50 ppm (probable phosphosugar on the basis of its multiplicity and chemical shift, neither glucose-1-phosphate nor glucose-1,6-bisphosphate by comparison with authentic standards). However, these unfitted peaks represent only a tiny fraction of the overall intensity. Finally, one sample in the 480 mg/kg dose group had a very high xanthine concentration (more than 20 times higher than the average for all other worms), which had high leverage in multivariate analyses, and so this one data point (not the whole sample) was treated as a missing value.

**Table 1:** List of NMR-visible metabolites assigned in earthworm extracts

Compound	Relative concentration^a^	Abbreviation	Number^b^	Functional class^c^
Acetate	10	Ac	1	Organic acid
Adenosine	9.3	Ado	2	Nucleoside
ADP	5.9	ADP	3	Energy
Alanine	62	Ala	4	AA
AMP	5.8	AMP	5	Energy
Asparagine	11	Asp	6	Basic AA
Aspartate	5.8	Asp	7	AA
ATP	0.76	ATP	8	Energy
Betaine	11	Bet	9	Membrane
Choline	2.9	Cho	10	Membrane
Dimethylamine	1.7	DMA	11	Other
N,N-dimethylhistidine	5.6	DMH	12	His compound
Fumarate	3.1	Fum	13	Organic acid
Glucose	27	Gluc	14	Sugar
Glucose-6-phosphate	0	G6P	15	Sugar
Glutamate	52	Glu	16	AA
Glutamine	34	Gln	17	Basic AA
Glycine	23	Gly	18	Membrane
HEFS	100	HEFS	19	Membrane
Histidine	2.4	His	20	His compound
Inosine	13	Ino	21	Nucleoside
myo-Inositol	12	m-Ins	22	Membrane
scyllo-inositol	9.9	s-Ins	23	Membrane
Isoleucine	6	Ile	24	Lipophilic AA
Lactate	47	Lac	25	Organic acid
Leucine	17	Leu	26	Lipophilic AA
Lombricine	78	Lom	27	Energy
Lysine	18	Lys	28	Basic AA
Malate	43	Mal	29	Organic acid
Mannose	3.3	Man	30	Sugar
Methionine	1	Met	31	AA
3-Methylhistidine	0.27	3MH	32	His compound
Nicotinate	2.9	Nic	33	Nucleoside
Phenylalanine	7.2	Phe	34	Lipophilic AA
Phosphoethanolamine	21	PE	35	Membrane
Succinate	15	Succ	36	Organic acid
Threonine	10	Thr	37	AA
Tryptophan	2	Trp	38	Lipophilic AA
Tyrosine	6.4	Tyr	39	Lipophilic AA
Uridine	1.3	Uri	40	Nucleoside
Valine	8.9	Val	41	Lipophilic AA
Xanthine	7.2	Xan	42	Nucleoside

^a^Mean value, expressed as a percentage of the most concentrated metabolite (HEFS).

^b^Number refers to label in Additional file 1.

^c^Corresponds to the labels in Figure 2. AA, amino acid.

**Figure 2:**
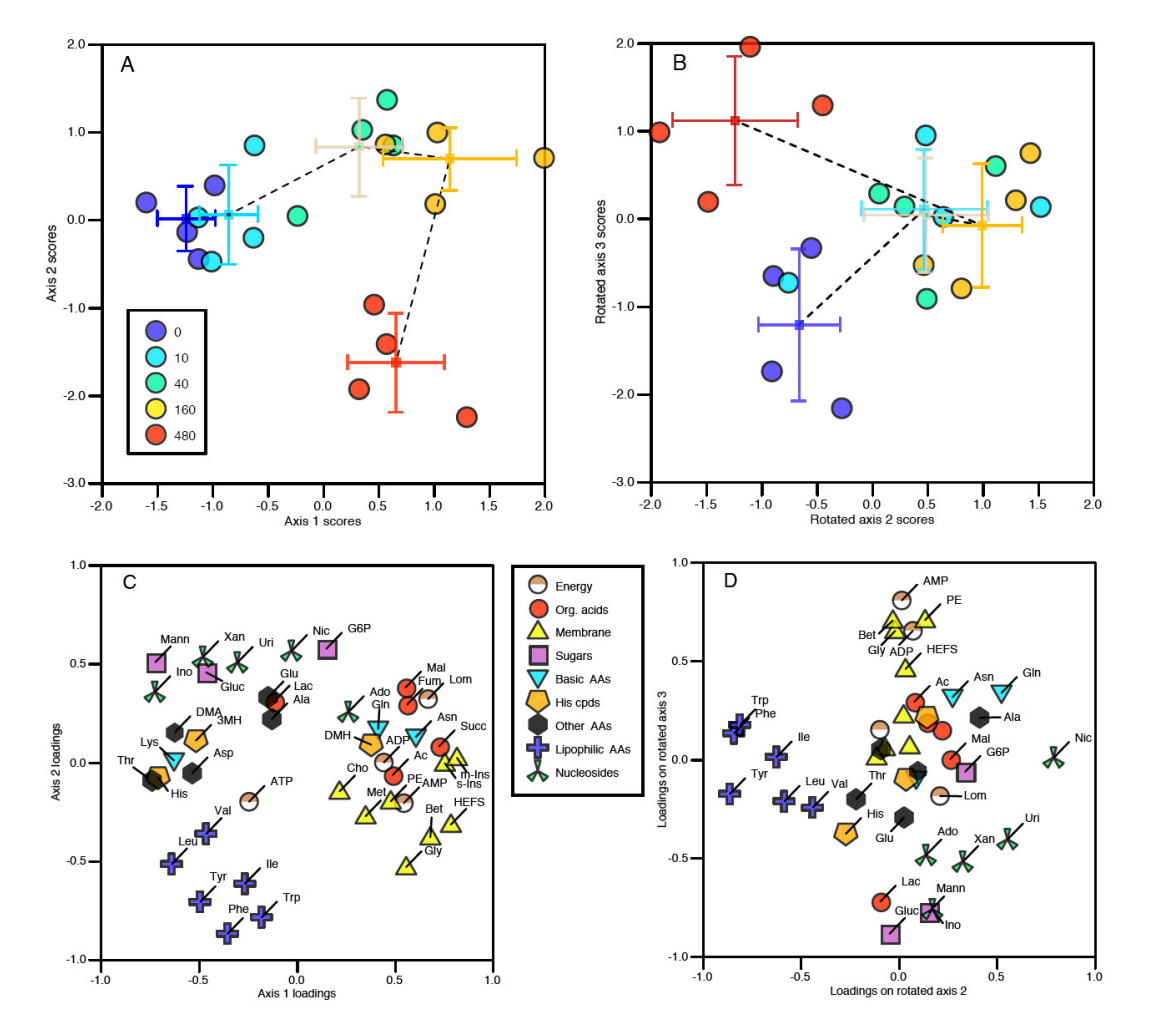
**Factor analysis of NMR spectral data showing relationship between metabolite profiles and copper exposure**. (A) Scores plot, axes 1 and 2. (B) Scores plot following Kaiser varimax rotation, axes 2 and 3. Data are shown for both individual samples and for dose group means ± standard deviation (SD); groups are joined in dose order by dashed line. (C) Loadings plot, axes 1 and 2. (D) Loadings plot following varimax rotation, axes 2 and 3. Loadings for individual metabolites are identified by abbreviations given in Table 1.

Factor analysis showed a clear relationship with copper on axes 1 and 2; in addition, it was obvious that at the highest-concentration dose (480 mg/kg soil) the worms were very different to the others along the second orthogonal axis (Figure 2). This was also true following varimax axis rotation, although, in this case, the copper-related differences dropped down to axes 2 and 3. HCA showed that the high-concentration samples (480 and 160 mg/kg) formed a separate cluster to the low-concentration samples (0 and 10 mg/kg), with the intermediate 40 mg/kg worms falling into both of these clusters (Figure 1). Given that not many samples were available for cross-validation, and that the metabolic responses to copper were clearly non-linear, we chose not to use additional supervised multivariate methods.

### Metabolic changes

There is currently no 'metabolite ontology' database that allows one to systematically annotate individual compounds into different groups; approaches based on network topology for the definition of elementary modes are exciting, but are still under development, and cannot be applied at the whole-organism level for metazoans [46]. Hence, we performed a simple annotation based on existing biochemical knowledge. We note that our annotations were not performed systematically; it is also important to realise that many metabolites should be annotated to multiple categories, which we did not attempt to do. Nevertheless, this simple approach may be a useful first step in interpreting metabolomics data.

Several metabolite groups exhibited a coordinated response; this was especially clear for the lipophilic amino acids (Figure 2). Several other possible coordinated responses were identified, including sugars, nucleotides, organic acids (including several synthesised through the citric acid cycle) and, possibly most interestingly, 'cell membrane compounds' (Figure 2). It should be noted that attempting to assign 'metabolite ontologies', at however limited a level, offers an option that simply does not exist to any great extent for genes: that of chemical similarity. (Some kind of chemical classification of genes might also be made, for example, on the basis of percentage occurrence of DNA bases, but this would be of very limited applicability.) Of the groups we have labelled in Figure 2, the 'cell membrane' group does not include any compounds that directly form cell membranes, but, instead, those that could be involved in either the anabolism or catabolism of lipid compounds. It should be noted that this is the most structurally diverse group that we labelled, containing an amino acid (glycine, which is a direct precursor to serine and, hence, sphingolipid metabolism, and is a breakdown product of choline and betaine), as well as metabolites that form polar lipid head groups (inositol compounds and phosphoethanolamine). In addition, we assigned the earthworm metabolite HEFS to this group: the very high tissue concentration of HEFS implies it has some kind of structural or stabilising role. As amphiphilic compounds are known to stabilise cell membranes during desiccation stress [47], and desiccation is a common and severe hazard faced by earthworms, we argue that the high HEFS concentrations probably reflect a role in membrane stabilisation.

**Figure 3:**
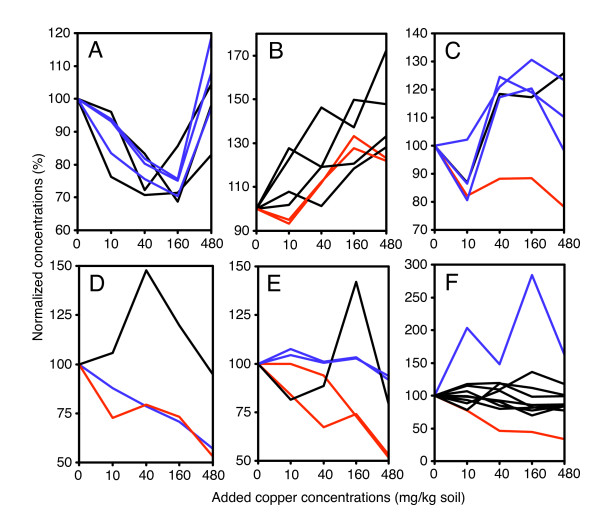
**Metabolite functional group responses to copper, concentrations expressed as a percentage of the mean control value**. (A) Lipophilic amino acids: black = aliphatic; blue = aromatic. (B) Cell membrane-related metabolites. Black = Bet, PE, Gly, HEFS; red = m-Ins, s-Ins. (C) Nucleosides. Blue = Nic, Uri; red = Xan, Ino; Black = Ado. (D) Organic acids. Blue = Mal, Fum, Succ; black = Ac; red = Lac. (E) Sugars and sugar phosphates. Blue = Mann; Red = Gluc; Black = G6P.

Having identified these potential groups through multivariate pattern-recognition analysis, we examined the actual data for coordinated functional group responses in more detail; selected groups are shown in Figure 3, which represents the percentage difference compared with controls (that is, directly equivalent to fold change). The coordinated response of the lipophilic amino acids is, again, very clear, and shows a non-linear response to copper: all of these metabolites are reduced at intermediate concentrations, and increased again at the highest level of 480 mg/kg (Figure 3A). In contrast, the membrane compound group shows a definite increase in response to copper (Figure 3B). The HEFS response also fits well within this group, supporting its classification as a 'membrane compound'. Even within these functional groups, fine-scale inter-metabolite relationships can be observed: *myo*-inositol and *scyllo*-inositol are the two most highly correlated metabolites within this group, with an apparently sigmoidal response to copper, presumably indicating metabolism through a common enzyme from lipid breakdown/turnover. The organic acids also show a sigmoidal-type relationship, with the compounds directly produced by the citric acid cycle the most closely related; lactate should probably not be considered part of the same functional group (Figure 3C). The remaining plots do not show grouping to the same extent: the sugars glucose and mannose both decrease in response to copper, but glucose-6-phosphate is not evidently correlated with these in any way (Figure 3D). Similarly, there is no obvious correlated response for the nucleosides as a group (Figure 3E); although nicotinate and uridine are both very highly correlated, the fold changes for these two compounds are very small, less than 10%. Larger changes are seen for inosine and xanthine, which decreased in response to copper; both of these are breakdown products, of purine and pyrimidine nucleotides respectively. Finally, the amino acids (excluding glycine and the lipophilic amino acids) showed little overall grouping. 3-Methylhistidine decreases in response to copper, while glutamine shows large variations (Figure 3F). In general, the fold changes are very small, with the large majority being less than twofold up or down. This contrasts with the transcript data, where larger fold changes are observed.

Non-targeted metabolite profiling has often been explicitly categorised as a biomarker discovery tool [48,49], with the implication that the aim of a metabolomic study is to discover a small number of biomarkers in amongst the biological noise. This approach may be advantageous when the desired outcome is a screening tool (for instance, for disease, or for environmental pollution), when a single (or few) robust biomarker(s) will enable the use of simpler and more robust predictive models (for example, classical linear discriminant analysis [49]) and targeted detection. An additional advantage not often made explicit is that this greatly enhances the transferability between different laboratories and, hence, the overall potential use and scientific value of the biomarkers. However, this approach is limited when seeking to integrate large datasets and to relate these to biological functions. In the current study, considering the coordinated responses of functionally related metabolites showed clear group responses, which would not have been obvious if only the most strongly changing metabolites had been investigated.

### Histidine compound metabolism

**Figure 4:**
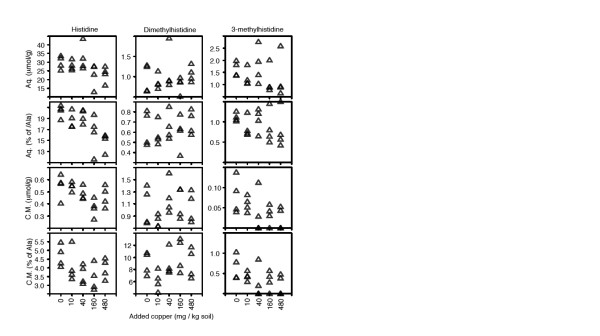
**Effects of copper on histidine compounds**. All values presented as either μmol metabolite per gram dry weight tissue or as relative concentration (percentage of alanine). 'Aq.' indicates sample extracted in water, allowing enzymatic proteolysis. 'C.M.' indicates sample extracted in chloroform/methanol, preventing further enzymatic activity.

Gibb et al [35] reported that histidine increased in earthworm tissue in response to copper contamination at levels comparable to the present study (up to 160 mg/kg soil), and speculated that this might represent a metabolite-level cellular response for direct detoxification of copper, as observed for the increase in free histidine seen in plant tissues in response to nickel [51]. In addition, histidine and DMH in *L. rubellus *were affected by metal contamination in worms sampled from the field [45]. We thus had a particular interest in histidine and related compounds, and examined their responses to copper in detail in addition to the general analysis above. We looked at both tissue and normalised concentrations (relative to alanine); it should be noted that the calculation of tissue concentrations assumed 100% extraction of metabolites and, hence, these values are likely to slightly underestimate true concentrations. There was a weak negative correlation with copper for histidine and 3-methylhistidine, and no apparent relationship with DMH (Figure 4). The earlier study used an aqueous tissue extraction [35], which would not prevent metabolic activity, especially as earthworm proteases are particularly active [52], and therefore their results may have included contributions from protein-bound histidine. It is certainly likely that a histidine-rich protein could be responsible for detoxification by binding copper [53]. Thus, we also extracted earthworm tissue in water, which was left at room temperature for 24 hours to allow any enzymatic activity to go to completion, and re-analysed the samples using NMR spectroscopy. Aqueous extraction causes extensive proteolysis, inferred from a large increase in free amino acid concentrations, which is effectively complete after one or two hours (data not shown). In general, the observed concentrations of amino acids were approximately two orders of magnitude higher than in chloroform/methanol extracts, and the separation of the different copper dose groups by factor analysis had completely disappeared (data not shown). Considering the histidine compounds only, there were essentially no dose-related differences compared with the chloroform/methanol extracts, indicating that there was no increase in protein-bound histidine that might reflect an increase in metal-binding proteins. We currently have no sure explanation of why our results are different to those previously observed. However, a note of caution has been sounded about the genetic variability of *Lumbricus *species used in laboratory tests [54], and we have previously observed high variability in histidine levels in *L. rubellus *that may have resulted from genetic differences between populations [45]. The population used by Gibb et al [35] may have had a different genetic background.

### Lipid analysis

**Figure 5:**
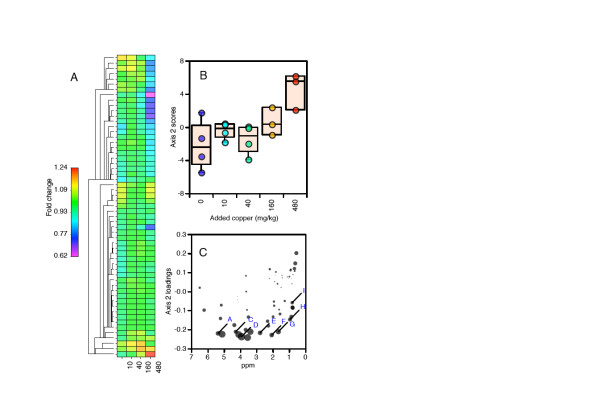
**Analysis of ^1^H NMR lipid data**. (A) Hierarchical cluster analysis of spectral regions (Euclidean distance) and heat-map showing fold-change relative to control group. (B) PCA, scores on axis 2. (C) PCA, loadings on axis 2. Area of point represents significance (-log(*P*), where *P *is probability from Student's *t *test; group 1, 480 mg/kg sample worms; group 2, 0, 10 and 40 mg/kg sample worms). Point labels in blue correspond to spectral regions shown in Additional file 7.

In addition to the polar fraction, we also analysed the total lipid fraction by ^1^H NMR. Lipid spectra are dominated by resonances from fatty acids; because these are all chemically similar, it is not usually possible to identify individual compounds by NMR, and thus it should be thought of as a way of obtaining information on the relative distribution of different moieties, which still constitutes useful biochemical information [55]. Given this, it was not possible to fit individual compound concentrations as for the polar compound data, and so we used a different approach for data analysis. A set of 57 integral regions was selected manually (Additional file 2); note that this does not equate to 57 separate compounds. A heat map of fold change relative to the control group shows quite clearly that the worms at the highest dose level, 480 mg/kg, are very different from the other groups (Figure 5). This is also visible on a single-sample (that is, not averaged) basis (Additional file 3). Factor analysis showed that one sample (one of the 480 mg/kg dose group) was an extreme outlier, which was also apparent on examining the original spectra, and so we excluded this sample and re-analysed the data. This showed clearly, using two different forms of scaling, that the 480 mg/kg worms were separated from the control and low-dose group worms along axis 2, while the 160 mg/kg worms were intermediate (Figure 5). Inspection of the original spectra confirmed that there were real and visible differences in spectral features (Additional file 4). Regions A and C-I represent signals that were decreased by copper exposure; A represents vinylic protons from unsaturated fatty acids, E represents protons allylic to two double bonds from polyunsaturated lipids and G probably represents protons from allylic methylenes that are not adjacent to another double bond. C represents glycerol peaks from triacylglycerols and D represents glycerol peaks from glycerophospholipids. F possibly represents plasmalogens, and H and I both represent signals from terminal methyls. (All assignments based on Sze and Jardetzky [55].) Region B contains a peak from an unassigned metabolite that is only present in the copper-treated worms, and is absent in the controls. Even though this peak is of very low intensity compared with the largest signals in the spectrum (for example, 0.0075% of the intensity of the main unsaturated methylene resonance following local baseline correction), it is a very specific response and so could still prove a useful future biomarker, possibly using some more sensitive detection method following compound identification.

### Microarray analysis

**Figure 6:**
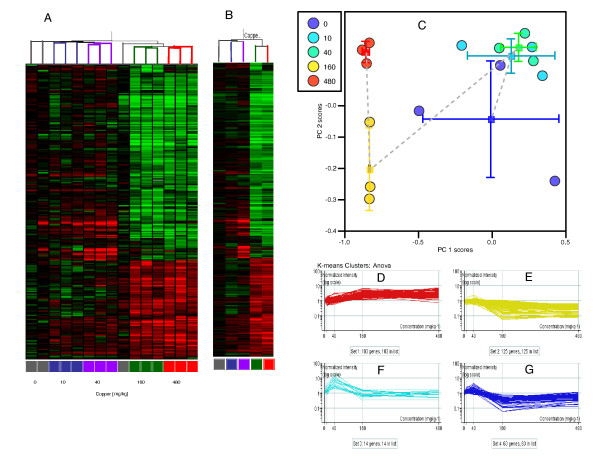
**Transcript profiles for copper exposed worms, 329 transcripts significantly differently expressed as a result of copper treatment (ANOVA)**. (A) Hierarchical cluster analysis and heat map, individual replicates. Colours of heat map indicate up-regulation (red) and down-regulation (green) of transcripts. (B) hierarchical cluster analysis and heat map, dose group averages. (C) PCA, first two components. (D)-(G) relative expression profiles (transcripts significantly different at the *p *= 0.05 level) fall into four clusters (*K*-means). Colours of different groups for (D)-(G) are for visualisation only.

There were 8,029 cDNA reporters on the microarray. Of these, 7,107 consistently yielded high-quality data and 1,705 exhibited a change in the level of transcription of more than twofold in one treatment group (Additional file 5). One-way analysis of variance (ANOVA), with correction of the error rate for multiple tests following the approach of Benjamini and Hochberg [56], revealed 329 of these transcripts as significantly altered (*p *< 0.05; see Additional file 6). Direct functional information relating to earthworm transcripts is negligible. However, approximately one-third display a significant homology (BlastX significance greater than 10^-10^) to human orthologues, and so we concentrated on these transcripts for further analyses. There were clear differences of expression in the different dose groups (Figures 6A and 6B). Pattern recognition analysis using principal components analysis (PCA) indicated that the major effect was at the two highest dose groups, which were clearly separated from the other samples (Figure 6C).

We used a *K*-means clustering approach to show that the transcripts fell into four response groups (clusters), which displayed similar dose-dependent profiles with respect to copper exposure. One group comprised of genes showing a general dose-dependent increase with respect to copper (Figure 6D), and two groups displayed the converse relationship being separated by the degree of down-regulation observed in the two lower copper doses (Figure 6E). The most intriguing cluster, however, were those transcripts that were up-regulated when exposed to a low dose of copper with a subsequent down-regulation at higher doses (Figure 6F). This profile indicates that the organisms' hormetic response to copper is also manifest at the level of transcription [57].

To complement the purely statistical approaches that provide insight into the coordination of transcript response, we also analysed the bias in gene function within transcripts (selected by unsophisticated filtering) to identify those that displayed more than twofold changes in transcript level in response to any of the copper exposures. We chose this group to be 'inclusive' because, although we recognise that it includes a small quantity of noise due to the quantity of transcripts analysed, it provided a more complete representation of functional group effects. This was especially desirable given that, because functional assignment was based on the top human orthologues, only about one-third of the reporters could be assigned confidently. We used a background created by those human orthologues assigned to the complete reporter set to calculate the representation bias for gene ontology terms for those orthologues assigned to transcripts exhibiting copper-induced twofold change in expression [58]. The ontology groups that were significantly overrepresented (*p *< 0.1) are summarised in Additional file 7. The clear disruption in genes associated with mitochondrial electron transport strongly implies a copper-induced mitochondrial dysfunction, consistent with previous observations in other organisms [59-61]. Even a slight reduction in the capacity for adenosine triphosphate (ATP) production by aerobic respiration would result in a redistribution of energy production through anaerobic processes, and this is indeed evident through the changes in genes involved with sugar mobilisation for energy production (Additional file 7). We also observed transcript changes that we interpret as representing functional interactions between copper and other essential metal ions, in particular changes in expression of calcium- and iron-binding proteins. Finally, there were clear alterations in lipid metabolism at the transcript level, complementing the observations made at the metabolite level.

**Figure 7:**
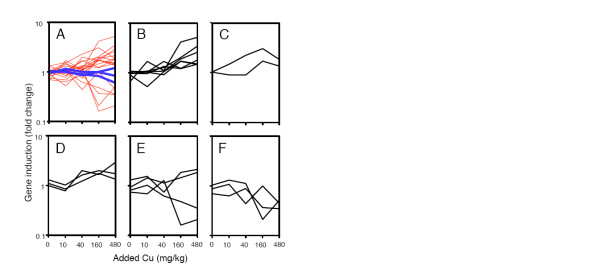
**Transcript profiles for copper exposed worms, transcripts selected on the basis of expected response to copper (that is, prior knowledge)**. All transcript levels are shown on a log_10 _scale ranging from 0.1- to 10-fold induction. (A) Invariant genes (blue) compared with all other selected genes (red). (B) Metallothionein genes. (C) Heat shock protein genes. (D) Genes involved in glutathione metabolism. (E) Genes involved in DNA repair mechanisms. (F) Regulators of apoptosis.

The power of calculating functional representational bias is inherent in the cumulative nature of the data obtained from a profiling approach. However, it is also important to extract specific transcript profiles of genes with an established and expected functional link to copper (Figure 7, and Additional file 8). For example, metallothioneins are widely responsive to metal exposure, including copper for particular isoforms, in a large number of organisms. Hence, we were interested in determining any such specific responses to copper in *L. rubellus*. The observed up-regulation of multiple reporters (Figure 7B) indicates that earthworm metallothionein, surprisingly [62,63], is induced by copper at a far lower exposure concentration level than by cadmium. Copper exposure also increased general toxic stress in the worms, as shown by the induction of HSP70 and HSP40 (Figure 7C); our observations here from the transcript profiles are consonant with previous targeted immunochemical analyses of HSP70 levels in earthworms exposed to copper and metalliferous soils [64,65]. Copper toxicity also causes an increase in generation of reactive oxygen species, as a result of the mitochondrial dysfunction discussed above. This could explain the increase we observed in specific glutathione-S-transferases (Figure 7D), also previously seen in response to copper toxicity [66]. These reactive oxygen species also damage DNA [67,68]. Similar DNA damage is also highly likely to have been a factor in the current study, as reflected by the alteration in levels of DNA repair enzymes and of enzymes implicated in cell cycle control, with both increases and decreases in transcript levels observed in response to copper (Figure 7E). Ultimately, copper-induced damage leads to apoptosis [69,70], which again is consistent with the transcriptomic data, with down-regulation of apoptotic regulators (Figure 7F). Summarising, the responses discussed here all represent plausible modes of cellular disruption, and all of them have been demonstrated previously to be induced by copper exposure. In sharp contrast, when we consider the response of a number of established control genes [71] and compare them with the other groups we have selected here, we see that they are not copper responsive (Figure 7A). This strongly supports the technical and biological validity of the transcript data. Further external validation is given by comparison with the metabolomic data, where the observations of metabolites are highly consistent with the metabolic changes suggested by the microarray data.

Samples for both microarray and metabolomic analysis were chosen by selecting worms with the best condition from each independent mesocosm, thus avoiding obviously diseased and/or parasitised worms. This has two major advantages for the molecular data. First, it avoids major confounding factors, for example, from chance-affected worms. Secondly, even in the case that there would be an interaction with copper treatment, for example, through copper toxicity decreasing the resistance to external factors, one would expect the molecular mechanisms induced to be general and stress-related. Thus, even though we may have selected individuals that did not provide the most accurate representation of ecological fitness under metal stress, the molecular endpoints will be more closely related to specific copper-induced mechanisms.

### Integration of the different datasets

There are multiple possibilities for integrating omic datasets, but these can be considered essentially to fall into two categories: statistical, that is, relying wholly on data-driven associations between variables (for example, [4,72]); and knowledge-based, that is, using prior knowledge about biological organisation and pathways/networks (for example, [73,74]). Clearly, an optimal solution would use information from both of these approaches; but then this, in the limit, effectively becomes a full model of metabolism, and this is not currently achievable, even for highly controllable unicellular model organisms. Here we have chosen a separate analysis of datasets and subsequent combination of observations.

**Figure 8:**
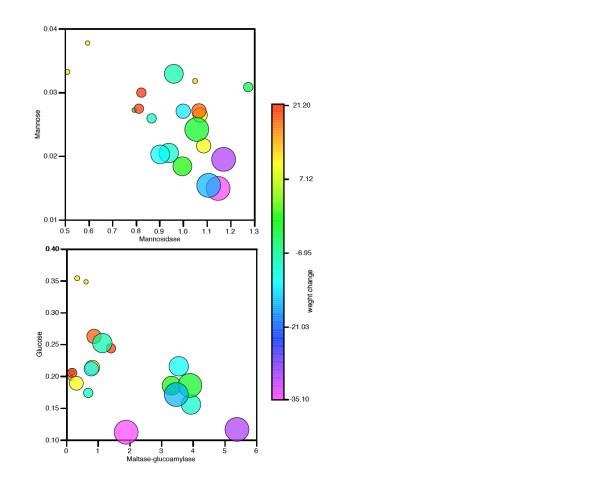
**Integration of transcript, metabolite and functional endpoints: relation to copper**. (A) Mannosidase versus mannose. (B) Maltase-glucoamylase versus glucose. For both, size of points represents added copper as ordered factor (that is, smallest points represent 0 mg/kg copper, largest points represent 480 mg/kg copper). Colour scale represents weight change (%), data taken from Spurgeon et al [37].

Specific genes that are up- or down-regulated were identified from the transcriptomic data (Table 2). Relatively few could be unambiguously annotated to metabolic enzymes; two of these are from carbohydrate metabolism, mannosidase and maltase-glucoamylase. Fortuitously, the metabolic substrate and product of these enzymes, respectively, could be identified in the NMR spectra. Figure 8 shows that in both cases there is a negative correlation between transcript and metabolite levels. This can be readily rationalised for the mannose/mannosidase relationship, that is, increase in the catabolic enzyme results in a decrease in substrate concentrations, but the opposite argument should hold for glucose/maltase-glucoamylase. This is a useful reminder that it may be dangerous to over-interpret metabolite pair relationships such as these, as the most important factor, metabolic fluxes, cannot be inferred directly from gene transcript levels or metabolite concentrations alone. More interestingly, these molecular data could be very clearly related to ecologically important endpoints. It is clear that a high-sugar/low-transcript level is, in both cases, associated with the low copper doses, and there is a distinct change in both metabolite and transcript concentration for the high-dose samples (Figure 8). Moreover, the worms have a positive energy balance for the first condition (low-dose/high-sugar/low-catabolic mRNA level), that is, they gain weight over the course of the experiment, but a negative energy balance, losing weight, for the second condition (high-dose/low-sugar/high-catabolic mRNA level).

**Figure 9:**
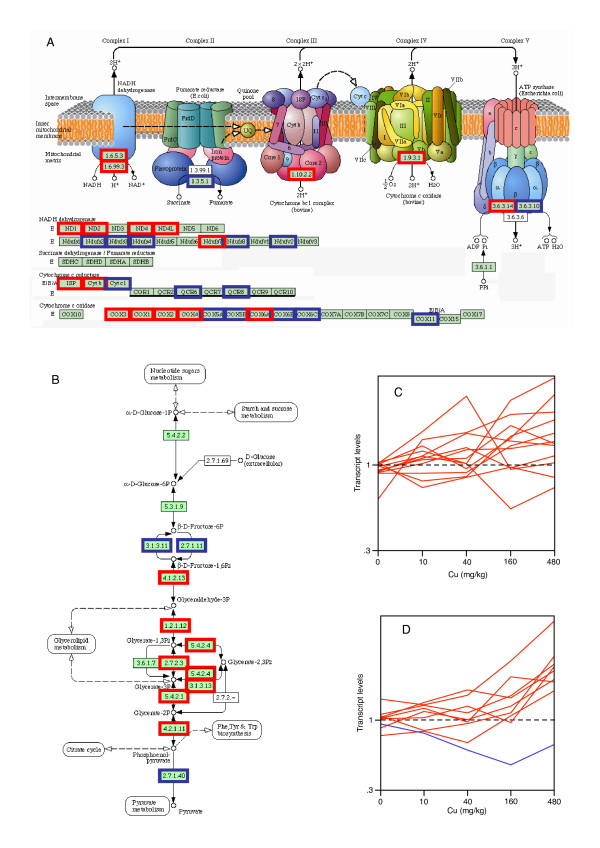
**Impact of copper on oxidative phosphorylation and on glycolysis/gluconeogenesis**. Human orthologues of transcripts exhibiting more than twofold alterations in their transcript level upon copper exposure were mapped onto the KEGG pathways [92] representing (A) oxidative phosphorylation (adapted from KEGG ID: hsa00190) and (B) glycolysis/gluconeogenesis (adapted from KEGG ID: hsa00010). Transcripts with more than twofold alterations are outlined in red, and those with less than twofold alterations are outlined in blue. Transcripts that are not outlined were not present in the gene set used for the cDNA microarrays. (C) The transcript levels on a log_10 _scale for the genes marked in (A). For genes represented by more than one transcript (multiple ESTs), mean values are shown. The black dotted line indicates no gene induction. (D) The transcript levels for the genes marked in (B) (red lines); phosphoenolpyruvate carboxykinase is also represented (blue line), although it is not shown in (B).

In addition, a more powerful approach than looking for alterations in *single *genes is to search for functional gene groups that are co-ordinately regulated. This is achievable for the transcript data, given that Gene Ontology (GO) classifications are available. Figure 9 shows that a number of transcripts represented with overrepresented ontology groups mapped onto specific Kyoto Encyclopedia of Genes and Genomes (KEGG) pathways, and it illustrated the impact of copper on genes involved in the electron transport pathway and converse impact on glycolysis, thus indicating the severe remodelling in energetic metabolism upon exposure to copper. The majority of transcripts for electron transport were up-regulated (Figure 9C), and this picture was even clearer for the glycolytic transcripts, which were all up-regulated (Figure 9D). Conversely, phosphoenolpyruvate carboxykinase (PEPCK) transcript levels were decreased by copper (PEPCK was a member of the GO class on which the analysis was based, even though it is not shown on the pathway fragment in Figure 9). Although this represents only one enzyme, it indicates that the gluconeogenic enzymes, as would be expected, were regulated oppositely to the glycolytic enzymes.

These observations are directly reflected by the metabolomic data, where a set of organic acids that are citric acid cycle intermediates were altered as a functional group, and there was a decrease in free sugar concentrations (Figure 3). In addition, when these observations are considered together with the links to specific transcripts and functional endpoints (Figure 7), and the changes in metabolites directly involved in maintaining cellular energy levels (*vide infra*), it is clear that energy metabolism in *L. rubellus *was profoundly impacted by copper treatment.

#### Interpretation of energy reserve data

Some copper-responsive metabolites were those related to maintenance of cellular energy levels, in particular adenosine phosphates. In general, adenosine monophosphate (AMP) and adenosine diphosphate (ADP) levels were both high and correlated to each other, and also showed some positive correlation to copper. This, combined with the lack of any detectable ^1^H signal for phospholombricine, implies that the worms' cellular energy reserves were exhausted, and thus not truly representative of *in vivo *metabolism. This is not a simple artefact of the extraction procedure: for comparison, a neutralised 6% perchloric acid extract tells a similar story about the energetic state, that is, AMP and ADP both higher in concentration than ATP, and also completely lacking a signal from phospholombricine (Additional file 9). The worms were sampled under conditions that would have preserved metabolic integrity, by flash-freezing into liquid nitrogen, storage at -80°C, and lyophilised tissues extracted directly into ice-cold solvent/water mixtures. In addition, the high quality of the mRNA extracted for the microarray analyses confirms the preservation of the biological integrity of the samples. Thus, we conclude that the general trend of the adenosine phosphates (AXP) data (ATP decreasing, and AMP and ADP increasing, with increased soil copper) is a reflection of a general disturbance in energy metabolism, as also shown by decreases in free sugars, and up-regulation of transcripts for breakdown of these sugars and catabolism of energy reserves, even if it may not be a true 'snapshot' of *in vivo *metabolism. Indeed, any extraction procedure is necessarily selective and different protocols will give different windows on the metabolome. Techniques such as high-resolution magic-angle-spinning of tissue biopsies (and even larger samples) are increasingly used for biochemical and biomedical investigations [75], even though this will lead to far more extensive enzymatic changes than the extractions used here.

### Sensitivity

There was a clear impact on both metabolomic and microarray profiles at the second-lowest additional copper concentration of 40 mg/kg. This is either as sensitive or more sensitive than the functional parameters measured, such as weight change, reproduction and the neutral red retention time (NRRT) bioassay, all of which had lowest-observed-effect concentrations (LOECs) of 160 mg/kg or higher, and much more sensitive than an endpoint based on mortality for which no significant effect was found at any of the tested exposure concentrations (that is, no-observed-effect concentration (NOEC) greater than 480 mg/kg) [37]. This sensitivity seems a fairly general rule, at least for laboratory experiments, where metabolomics has been shown to be extremely sensitive to individual chemicals compared with traditional endpoints [76]. Such sensitivity provides an interesting insight into the likely value of such analyses for regulatory testing regimes. Here omic approaches may provide insights on low effect level metabolic dysfunctions which may have implication over a life-time exposure, but may not be revealed in the time-limited chronic bioassays that form a cornerstone of modern chemicals policy [77].

Like some of the observed phenotypic responses (for example, reproduction rate and weight change), the omic data (both transcripts and metabolites) very clearly demonstrate a non-linear response to copper. This was also true for the weight change data, with an initial increase in body weight followed by a clear decrease at higher concentrations. It has been argued that hormesis is a very general response to potential toxins, with low-level exposure either being beneficial or stimulating a compensatory response [57]. While the response seen for copper may merely represent the fact that this metal is both essential and toxic for earthworms, it is currently the case more generally that a mechanistic basis for understanding such 'hormetic' responses is lacking. While initial suggestions have been made to explain hormesis in terms of an overcompensation of homeostasis to disruption that is mediated by the different affinities of stimulatory and inhibitory regulatory pathways [78], such suggestions have yet to be confirmed or refuted in empirical studies. Our data clearly show that the hormetic response in this case is manifest at a molecular level, indicating a possible role for metabolomics in understanding the mechanistic nature of the response.

## Conclusion

Drawing reliable inferences from omic data is often difficult, especially for non-model organisms which may be less well annotated than standard laboratory models. Here we have used a dual metabolomic and transcriptomic strategy, with each element providing complementary data, thus reinforcing the inferences made from the independent datasets. Interpreting the data in terms of functional groups, for both transcript and metabolite data, showed clear molecular group responses to copper. The integrated dataset indicated a clear alteration of energy metabolism as a result of sub-lethal copper exposure, with an increased switch to metabolism of stored carbohydrates, presumably as a consequence of copper interfering with mitochondrial function and reducing the amount of energy available from oxidative phosphorylation. These molecular impacts resulted in higher-level endpoints, including a reduction in body weight at high copper levels, reflecting the changes in energy metabolism, and a decrease in lysosomal integrity, indicating effects on membranes that could parallel the observed impacts on mitochondrial function.

## Methods

### Exposure and sampling

A full description of the earthworm exposure and sampling conditions is given by Spurgeon et al [37]. Briefly, worms were exposed for 70 days under field conditions in mesocosms to controlled levels of soil copper (0, 10, 40, 160 and 480 mg additional copper per kilogram dry weight soil; original copper content of the soil was 16.1 mg/kg), with four replicate mesocosms per dose level. A number of functional endpoints were measured including survival, weight change (average value per mesocosm), NRRT and cocoon production rate. Fifteen adult worms were exposed per mesocosm, and at the end of the experiment, three worms were used to measure tissue copper concentrations; four worms were used to measure NRRT and the three worms maintaining highest condition in each replicate were pooled and used for both transcriptomic and metabolomic analysis. This avoided inclusion of diseased or heavily parasitised worms within the omic experiments that may have otherwise confounded results. The worms were flash-frozen in liquid nitrogen, and ground to powder in a mortar and pestle under liquid nitrogen to obtain a single sample for each mesocosm. Total mRNAs from the samples were then isolated using established protocols [79,80]. The remaining sample was lyophilised without allowing the sample to thaw, and stored at -80°C until extracted for metabolomic analysis.

### cDNA microarrays

The *L. rubellus *EST project [80] has established a database of over 17,000 ESTs from unexposed and chemically exposed earthworms (see [82] for details). All sequences were assembled into clusters and annotated using the PartiGene pipeline [83]. To fabricate the glass slide cDNA microarray, a representative EST (usually the longest) was selected from each of the 8,029 clusters assembled from the ESTs (see [82] for full details). This sequence was polymerase chain reaction (PCR)-amplified and aliquots (5 μl) of concentrated products mixed in 384 well plates with an equal volume of dimethyl sulphoxide (DMSO). These were then printed onto Ultra-GAP glass slides (Corning) using 48 SMP3 pins (Telecham) mounted in a Spotarray 72 (Perkin-Elmer). Landmarks were introduced at the left-hand corner of each sub-array by the introduction of five replicates of the Lucida Scorecard (Amersham) gene reporters, which show no cross-reactivity to earthworm transcripts. All reporters were cross-linked to the slide by baking at 80°C for 2 hours, and UV cross-linking.

Lucida Scorecard test spike (Amersham Life Sciences) was added to 10 μg of total RNA prior to oligo-dT reverse transcription and coupling to Cy3 using an indirect amino amyl procedure. Clean-up of labelled targets, yield and integrity were all measured according to Owen et al [82]. A reference design was used in which approximately 30 pmol of Cy3 labelled target RNA was hybridised against the common oligonucleotide reference (representing 30 pmol of Cy5). The reference used was a 65–70 mer oligonucleotide designed against the vector sequence between the amplification primer binding site and cDNA insert. Use of this universal reference design allowed a comparison of slides to be made both within and between experiments. After hybridisation (18 hours), slides were washed and imaged according to Owen et al [82]. Array images were subjectively quality controlled for artefacts that would compromise quantification such as background effects and spot morphology prior to image analysis with Imagene 5.0 (Biodiscovery). Subsequently, the calibration standards from the Lucida Scorecard were analysed to objectively assess the sensitivity range and to define both saturation and background readings (Additional file 10). The microarray data can be accessed through the NEBC file store [84] (EnvBase accession number [EGCAT:4024]).

### Statistical analysis of microarray data

Numeric data were imported into LimmaGUI [85,86] which allowed the subtraction of background measurements, generation of additional quality control plots and subsequent normalisation using a within-array Tiplowess manipulation. Abnormally distributed samples were excluded from further analysis. Normalised data were subsequently imported into GeneSpring 7.3 (Agilent Technologies, Stockport, UK) and represented relative to the median expression within the control group. Final, quantity assessment was performed by visualising a box plot of the normalised data together with the generation of MA plots for the average data from each dose (Additional file 11). Abnormally distributed samples were excluded from the analysis.

Using GeneSpring, the three control slides and three slides for the exposure concentration that most closely matched the cocoon production EC_10 _for each chemical were used to identify chemically responsive genes. The data were first filtered to include only spots flagged as present in 3 out of the 15 slides. This filtered dataset was then used to generate three gene lists for each dataset. These were: (1) genes with a more than twofold difference in mean expression between control and exposed samples; (2) genes with significantly different (*p *< 0.05) expression between control and exposed samples after correction for multiple sample testing [56].

### NMR spectroscopy

Tissue samples (20–30 mg) were homogenised into 3 ml of extraction solvent (Heidolph SilentCrusher S) using a modified Bligh and Dyer protocol [87,88], in which a monophasic chloroform/methanol/water extraction is followed by the addition of water and chloroform, splitting the sample into two phases. The proportion of water was adjusted for the fact that lyophilised tissue was used, assuming a 90% water content in normal tissue. Both fractions were dried at 40°C using a rotary vacuum concentrator. The polar fraction was then resuspended in 0.65 ml of NMR buffer (100 mM phosphate buffer pH 7.0 in ^2^H_2_O, also containing 0.98 mM sodium trimethylsilyl-2,2,3,3-^2^H_4_-propionate (TSP); the ^2^H_2_O provided a field frequency lock for the spectrometer and reduced the signal from water protons) and centrifuged (5 minutes, 16,000 g). The supernatant (0.6 ml) was then transferred to 5 mm NMR tubes. The aqueous samples were analysed at 300 K on a 14.1 T DRX600 Avance NMR spectrometer (Bruker BioSpin, Rheinstetten, Germany) with a 600 MHz proton resonance frequency, equipped with a 5 mm broadband inverse probe. The samples were run using an automatic tube changer, over a period of about 12 hours, during which time they were kept at room temperature; the samples were loaded onto the tube changer in randomised blocks. A one-dimensional NOESY sequence with a mixing time of 100 ms was used for water suppression of the residual HOD in the NMR buffer, using a 30 Hz presaturation pulse. The spectra were acquired for 128 transients, with four dummy scans, into 32 K data points over a 12 kHz spectral width. A 3.5-second longitudinal relaxation recovery delay was added for each transient, giving a recycle time of 5 seconds. The chloroform fraction was resuspended in 0.65 ml of CDCl_3 _containing 0.03% tetramethylsilane (TMS) and transferred to 5 mm NMR tubes. The lipid samples were then analysed using a Carr-Purcell-Meiboom-Gill (CPMG) sequence, with a 8.65-second longitudinal relaxation delay giving an approximately 10-second recycle time (the CPMG sequence gave improved baselines and slight reduction of broad resonances compared to a simple pulse-acquire experiment). The raw data (free induction decays (FIDs)) for all spectra, and the fitted compound data for the polar extracts (*vide infra*), are available through the NEBC file store [84] (EnvBase accession number [EGCAT:4024]). The fitted compound data are also provided as Additional file 12.

### NMR processing and data analysis

The spectra were initially processed using iNMR 2.2.7 (Nucleomatica, Molfetta, Italy). The summed FIDs were multiplied by an exponential window function equivalent to 0.5 or 1 Hz line broadening (aqueous and lipid spectra, respectively). They were then zero-filled by a factor of 1.5, and Fourier transformed. Phasing was carried out using the automatic 'metabolomic phase correction' option, and adjusted manually where necessary; baseline correction was performed using an automatic first-order polynomial fit. All spectra were referenced to TSP/TMS at 0 ppm. The spectra from polar (aqueous) samples were further analysed using Chenomx NMR Suite 4.6 (Chenomx, Edmonton, AB, Canada). This software fits idealised spectra made up of combinations of Lorentzian line shapes, based on spectra of authentic standards, and estimates compound concentrations using TSP as an internal quantitation standard. Compounds were fitted using the proprietary Chenomx 600 MHz library, to which we added standards for glucose-6-phosphate, HEFS and lombricine. HEFS and lombricine are not commercially available; we purified HEFS from earthworm tissues using solid-phase extraction with a mixed-mode C18/anion exchange phase. Only a single peak for lombricine was fitted, based on a spectrum of an existing tissue extract acquired under fully relaxed conditions, and concentration estimated by comparing this with the integral of a known concentration of TSP. Thus, it is likely that the absolute accuracy of the lombricine concentrations will be lower than for the other compounds reported in this study, although the relative levels (precision) will be comparable. The fitted compound concentrations are available in Additional file 12.

For pattern recognition, data were normalised following the method of Dieterle et al [89] in which each profile is compared with a representative reference (in our case, a median of all sample profiles). The relative fold change for each variable in turn is calculated, and all values for a spectrum are then divided by the median fold change for that spectrum. Data were log-transformed by log(*n*_*i *_+ 0.018) for concentrations (expressed as mM); the value of 0.018 was chosen because it effectively removed the correlation between intensity and standard deviation for a series of five technical replicates, that is, increasing homoscedasticity (the principle is discussed elsewhere [90,91]). Factor analysis and hierarchical cluster analysis on non-centred data was carried out using Aabel 2.2 (Gigawiz, Tulsa, OH, USA). (Note that the factor analysis here is exactly equivalent to PCA carried out using the correlation matrix, but we have retained the term 'factor analysis' for consistency with the terms used in the software package.) The lipid spectra were integrated within selected regions using iNMR (integral boundaries given in Table 2).

## Authors' contributions

JGB carried out NMR spectroscopy, metabolomic data analysis, and drafted the manuscript. JKS developed experimental methods. FR carried out spectroscopic analysis of lipid samples. DJS and CS were responsible for earthworm exposure, sampling and functional assays. JW carried out hybridisations and microarray analysis. PK was responsible for analysis of microarray data and integrative pathway-level analysis. DJS, CS, SRS, AJM and PK participated in conception and design of the study. All authors read and approved the final manuscript.

## Supplementary Material

Additional file 1 A 600 MHz ^1^H NMR spectrum of typical earthworm extract, polar fraction. (A) and (B) have an expanded vertical scale compared with (C) and (D). Resonance from HEFS (compound 19) at 6.19 ppm is not represented at its full height. Metabolite labels correspond to numbers given in Table 1. * represents an unknown compound that is a probable breakdown product of HEFS. Click here for file

Additional file 2 Chemical shift regions for integrals of lipid extract spectral data ('missing' int01 was for internal standard TMS). Click here for file

Additional file 3 NMR integrals of lipid data, heatmap showing individual replicates. Click here for file

Additional file 4 600 MHz ^1^H spectra of lipid extracts. Samples only shown from control (blue) and highest (red) dose groups. One spectrum from red group was excluded as an outlier and is not shown here. (A) Vinylic protons from unsaturated fatty acids; (B) unassigned; (C) glycerol protons from triacylglycerols; (D) glycerol peaks from glycerophospholipids; (E) protons allylic to two double bonds; (G) protons allylic to one double bond; (H) and (I) terminal methyls. Click here for file

Additional file 5 Table of transcripts showing more than twofold change in expression as a consequence of copper exposure. Click here for file

Additional file 6 Table of transcripts significantly changed in earthworms exposed to copper. ANOVA, *p *< 0.05, Benjamini and Hochberg [56] FDR correction. Click here for file

Additional file 7 Table of GO terms overrepresented in transcripts whose expression is altered by copper exposure by more than twofold. Click here for file

Additional file 8 Table of the targeted functional transcript changes observed during copper exposure of adult earthworms. Click here for file

Additional file 9 Comparison of 6% perchloric acid (red) and chloroform/methanol (blue) extraction methods for earthworm tissue: (A) aromatic region; (B) aliphatic region. Click here for file

Additional file 10 Assessment of micro-array sensitivity and signal linearity. Representative analysis of the fluorescent signal generated by 10 RNAs introduced at known concentrations prior to labelling and detected by complementary reporter (10 replicates of each reporter spotted on the array). Data were generated from representative arrays selected from transcript analyses performed on RNA extracted from control and copper-dosed samples. (A)-(E) represent data from copper exposures of 0, 10, 40, 160 and 480 mg/kg, respectively. The average signal is indicated by closed circles with technical error bars representing the standard error of the measurements. A fitted regression line is shown for the linear portion of the response together with the *R*^2 ^value for the fitted line. Click here for file

Additional file 11 Graphical representations of relative gene expression against fluorescence intensity from control and copper-exposed samples. Array data were normalised and filtered (as described in Methods) and the log_2 _of the average fold change (M) plotted against the log_2 _of the average mean signal intensity (A). (A)-(E) represent data from copper exposure of 0, 10, 40, 160 and 480 mg/kg, respectively. Click here for file

Additional file 12 Table of metabolite concentrations as determined by 600 MHz ^1^H spectroscopy (μmol/mg tissue dry weight). Click here for file
