# Supplementary figures and images for: 'Systems toxicology' approach identifies coordinated metabolic responses to copper in a terrestrial non-model invertebrate, the earthworm Lumbricus rubellus
Source: BMC Biol. 2008 Jun 3;6:25. doi: 10.1186/1741-7007-6-25 (PMC2424032; doi:10.1186/1741-7007-6-25)

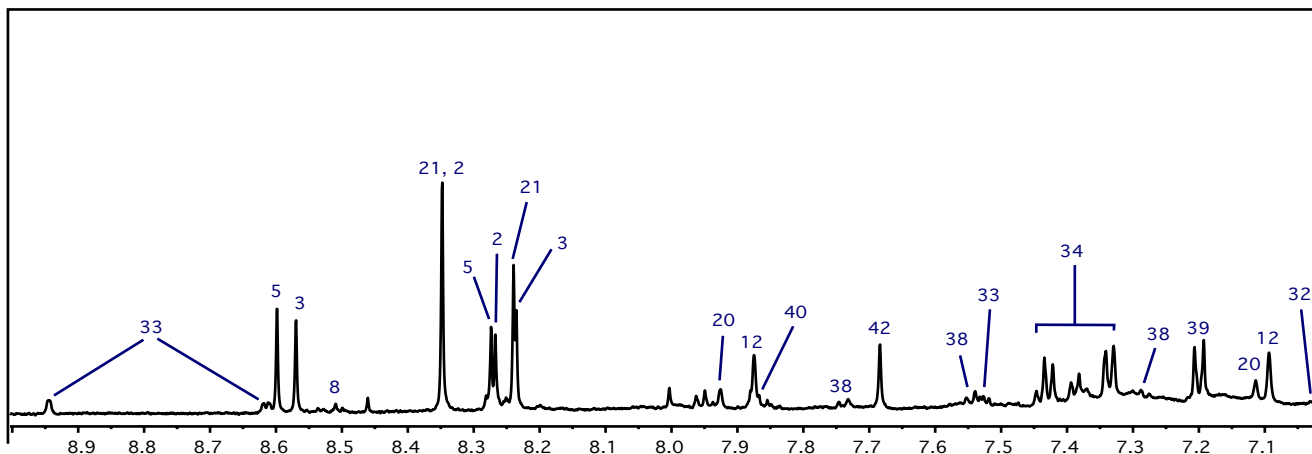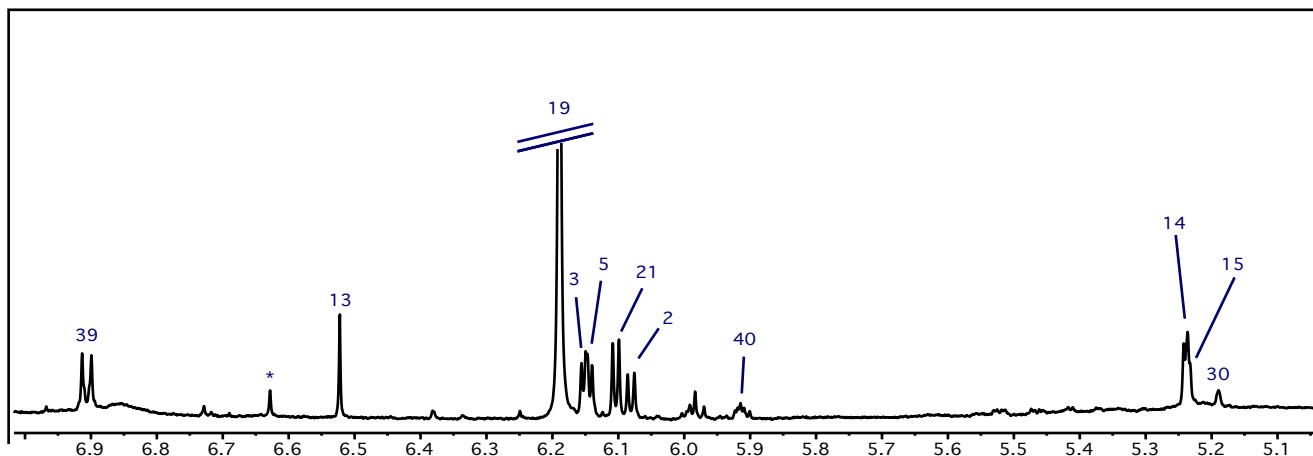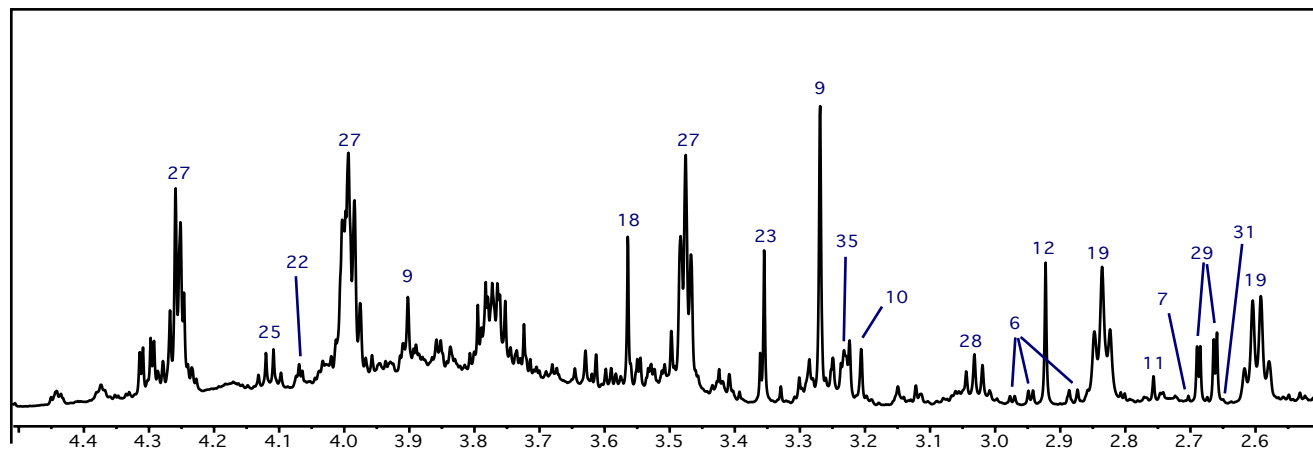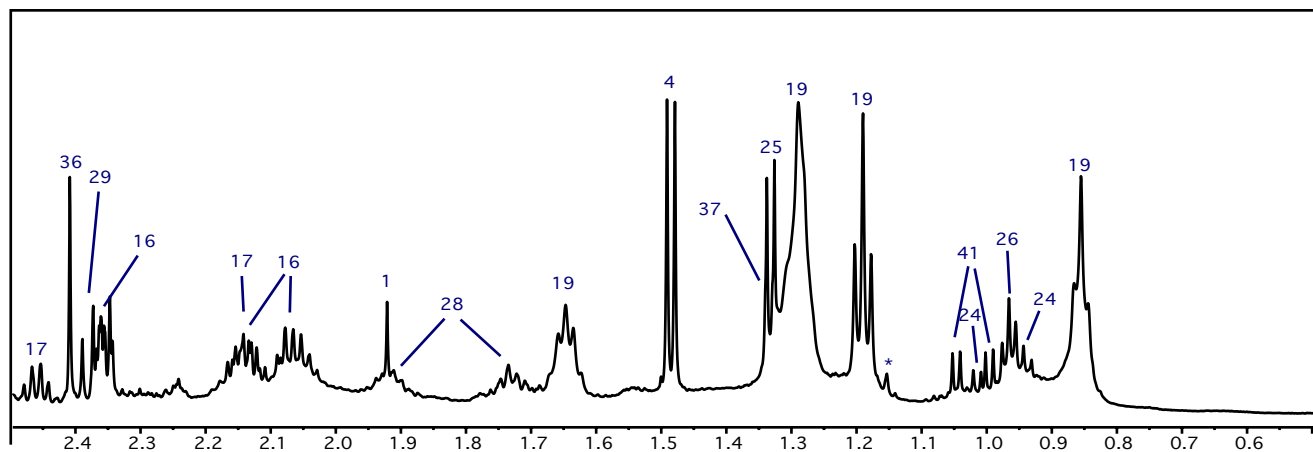

Supplement: Additional file 1 — A 600 MHz 1H NMR spectrum of typical earthworm extract, polar fraction. (A) and (B) have an expanded vertical scale compared with (C) and (D). Resonance from HEFS (compound 19) at 6.19 ppm is not represented at its full height. Metabolite labels correspond to numbers given in Table 1. * represents an unknown compound that is a probable breakdown product of HEFS. [file 1741-7007-6-25-S1.pdf]

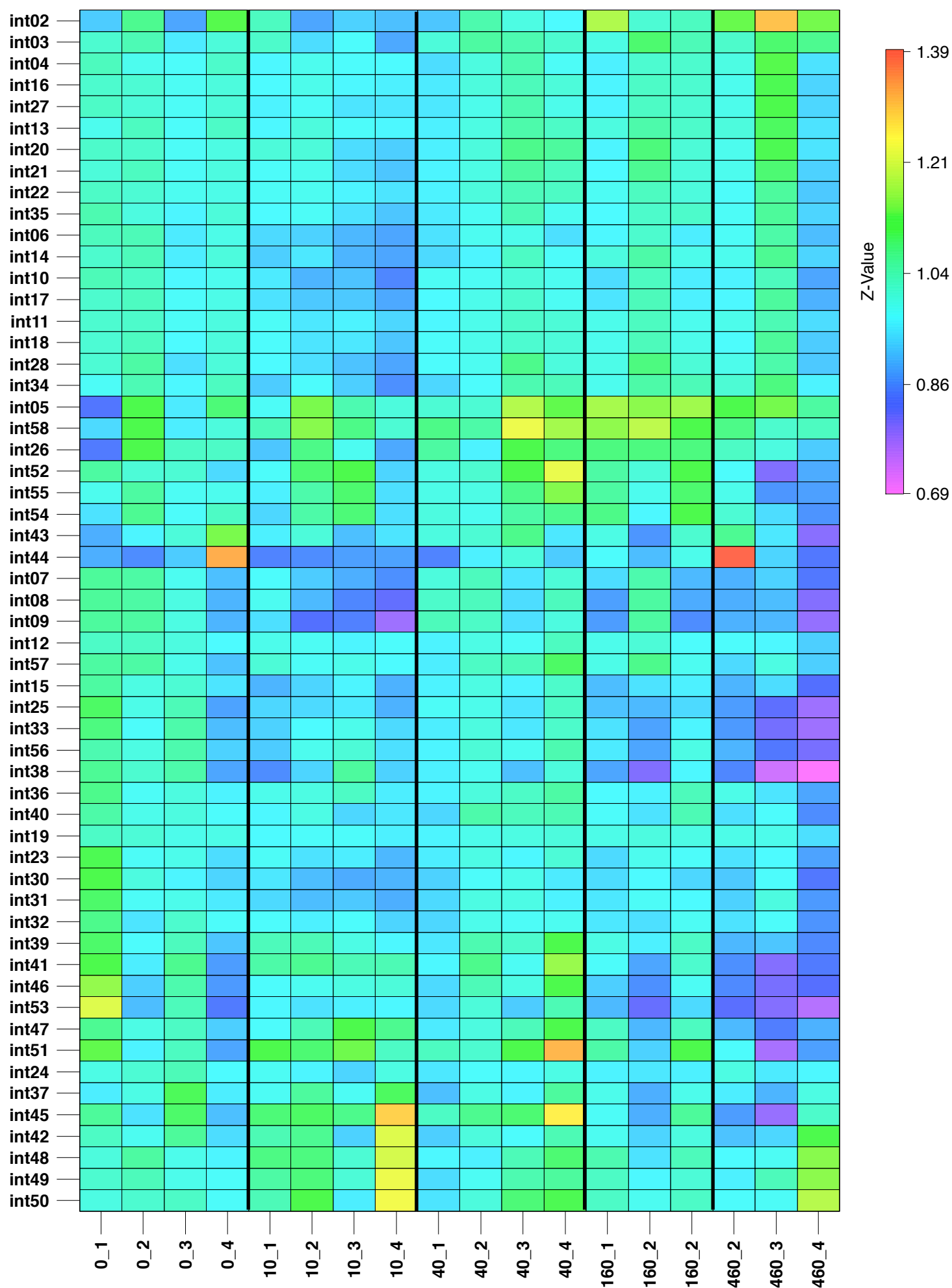

Supplement: Additional file 3 — NMR integrals of lipid data, heatmap showing individual replicates. [file 1741-7007-6-25-S3.pdf]

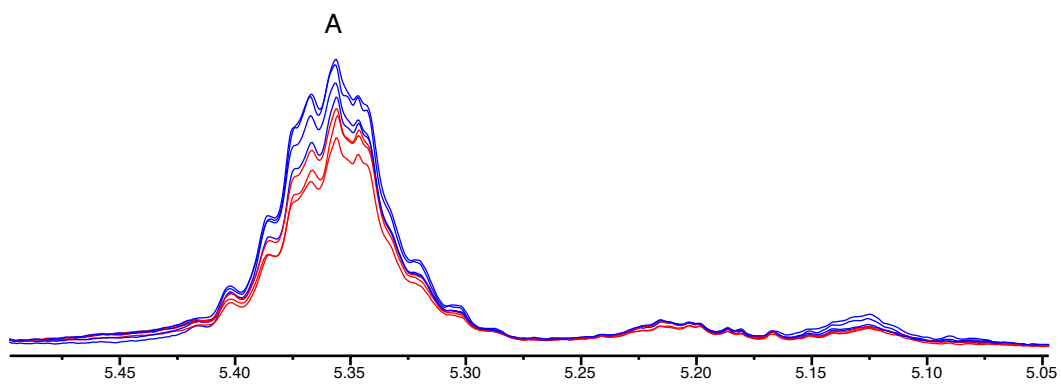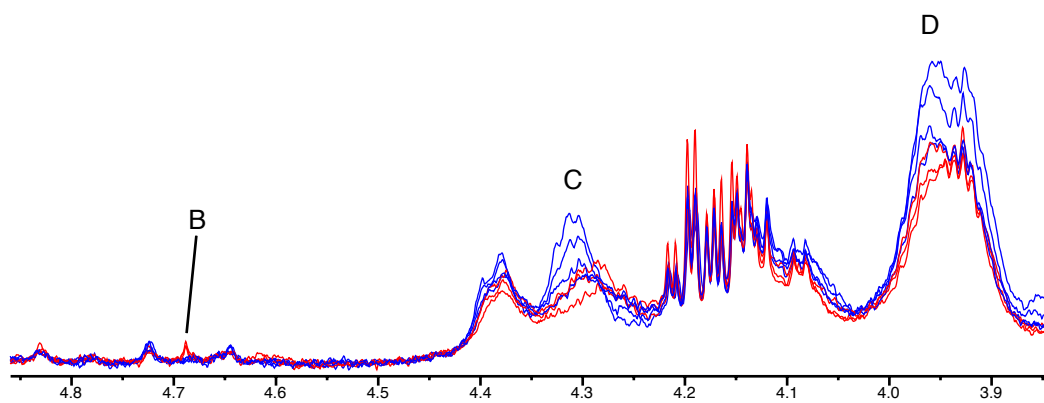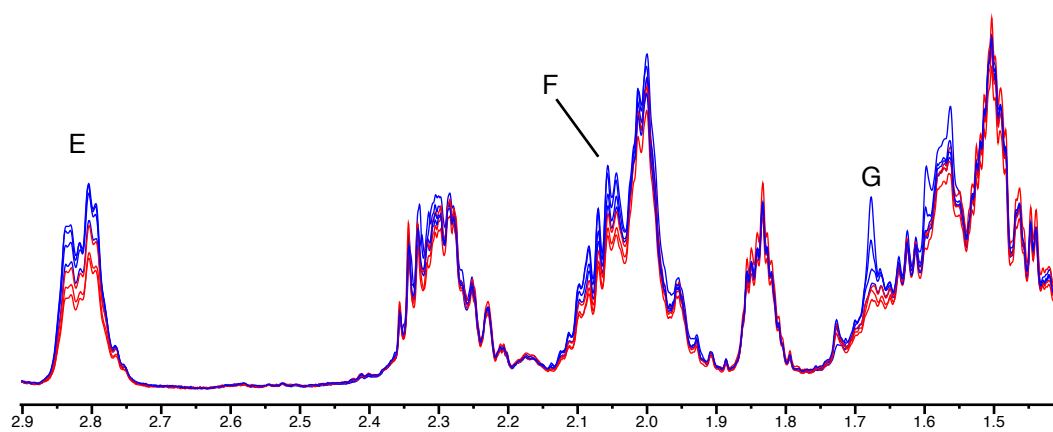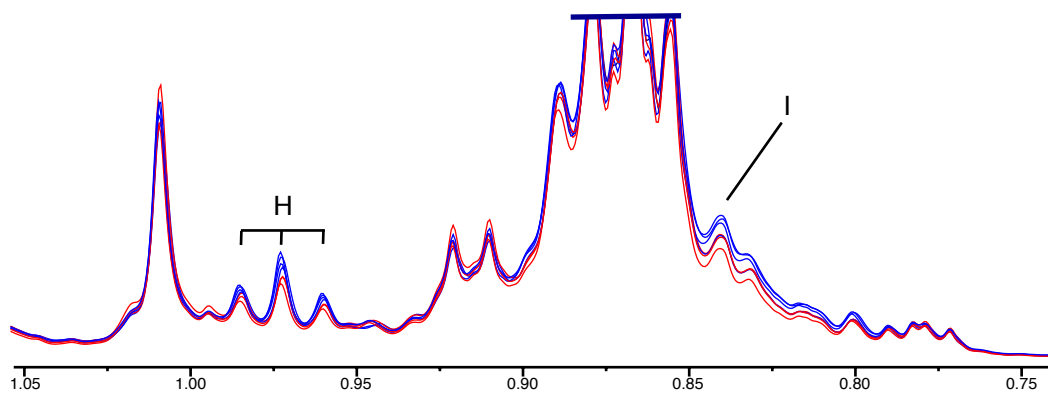

Supplement: Additional file 4 — 600 MHz 1H spectra of lipid extracts. Samples only shown from control (blue) and highest (red) dose groups. One spectrum from red group was excluded as an outlier and is not shown here. (A) Vinylic protons from unsaturated fatty acids; (B) unassigned; (C) glycerol protons from triacylglycerols; (D) glycerol peaks from glycerophospholipids; (E) protons allylic to two double bonds; (G) protons allylic to one double bond; (H) and (I) terminal methyls. [file 1741-7007-6-25-S4.pdf]

A

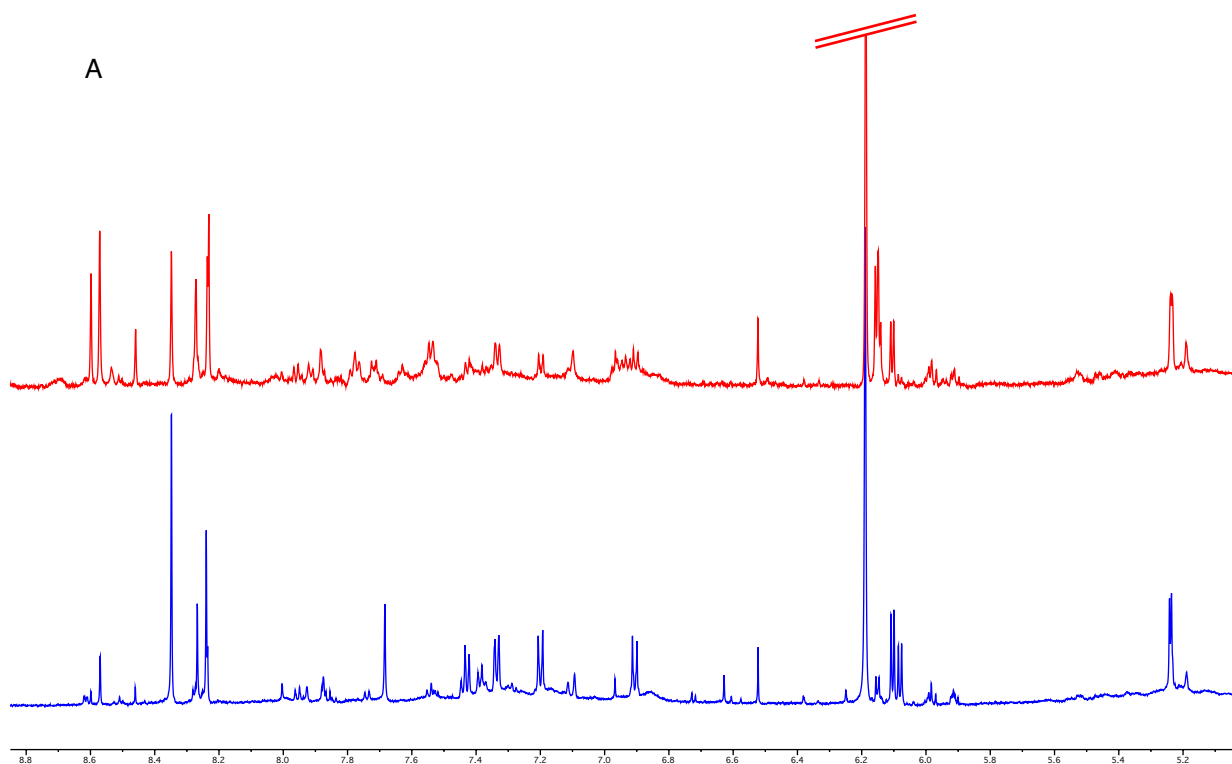

B

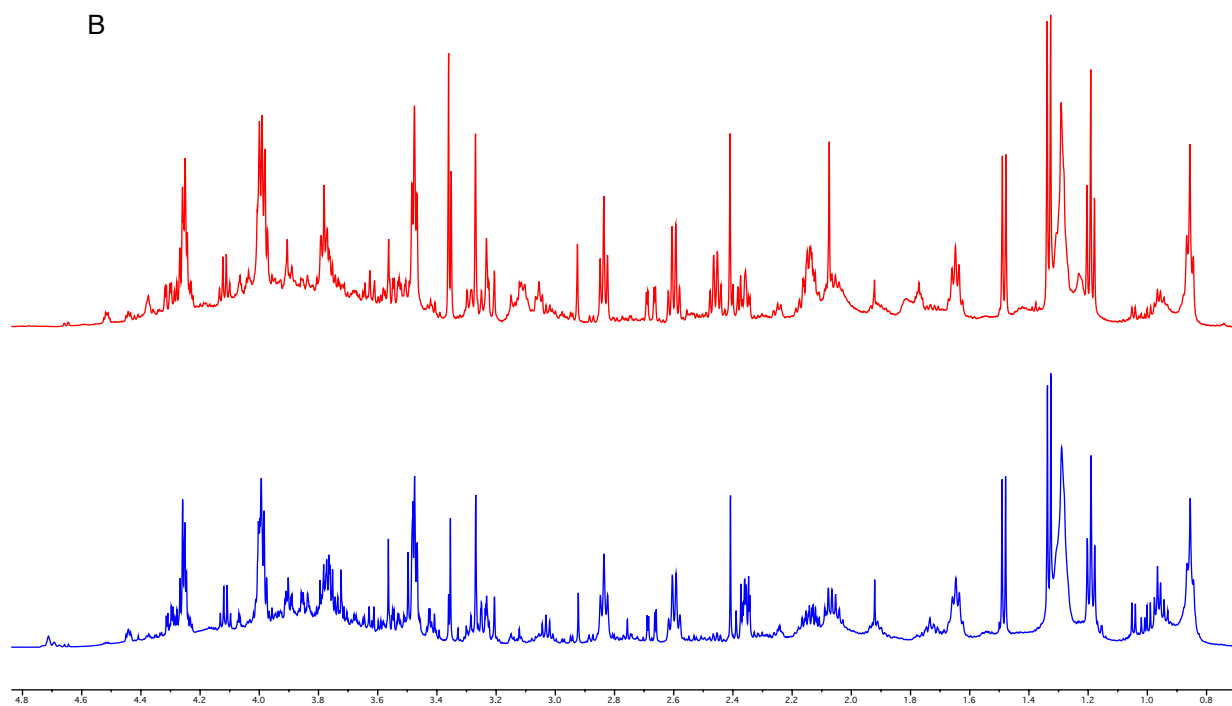

Supplement: Additional file 9 — Comparison of 6% perchloric acid (red) and chloroform/methanol (blue) extraction methods for earthworm tissue: (A) aromatic region; (B) aliphatic region. [file 1741-7007-6-25-S9.pdf]

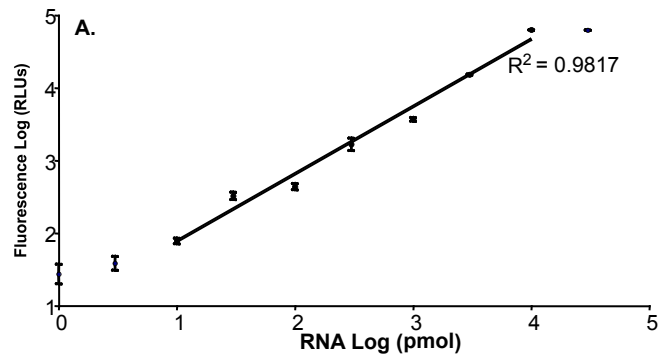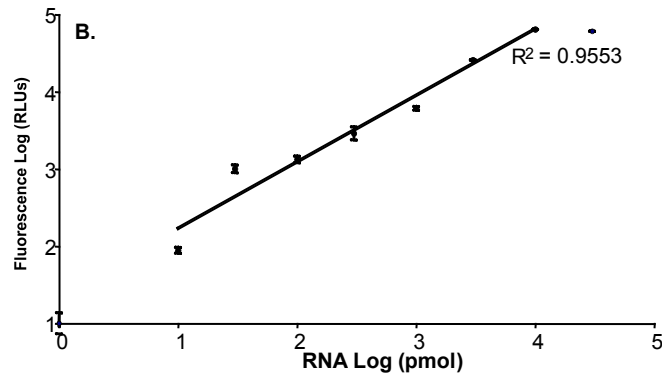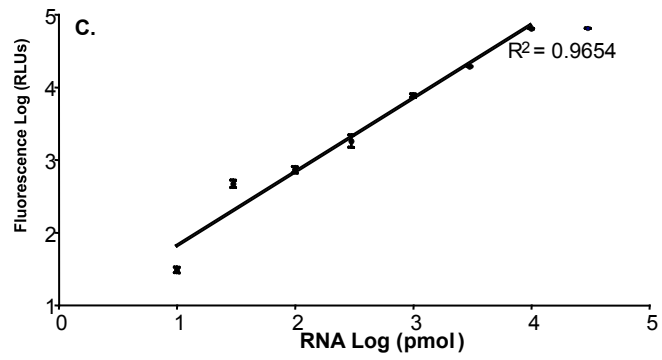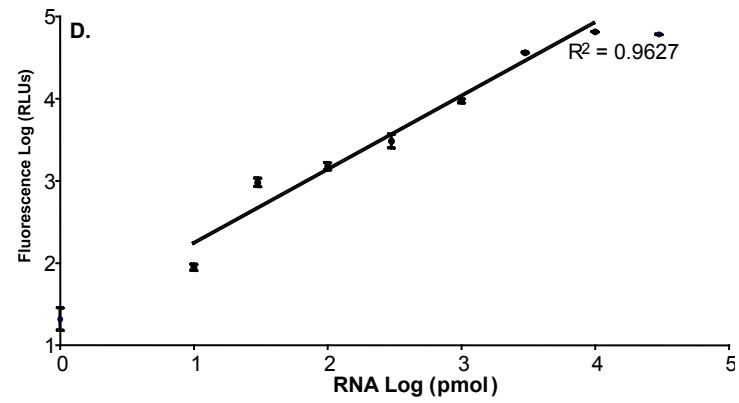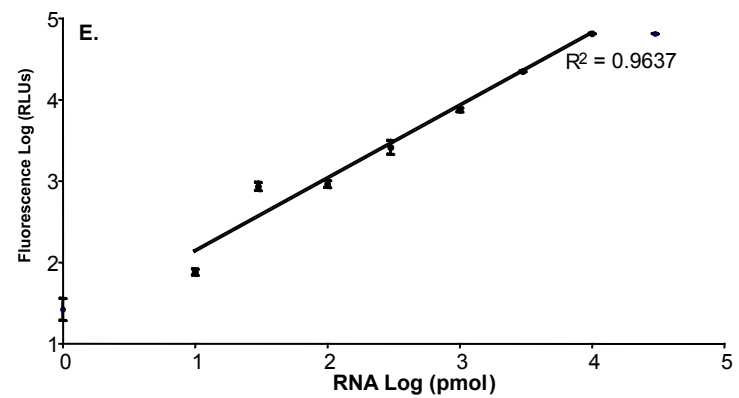

Supplement: Additional file 10 — Assessment of micro-array sensitivity and signal linearity. Representative analysis of the fluorescent signal generated by 10 RNAs introduced at known concentrations prior to labelling and detected by complementary reporter (10 replicates of each reporter spotted on the array). Data were generated from representative arrays selected from transcript analyses performed on RNA extracted from control and copper-dosed samples. (A)-(E) represent data from copper exposures of 0, 10, 40, 160 and 480 mg/kg, respectively. The average signal is indicated by closed circles with technical error bars representing the standard error of the measurements. A fitted regression line is shown for the linear portion of the response together with the R2 value for the fitted line. [file 1741-7007-6-25-S10.pdf]

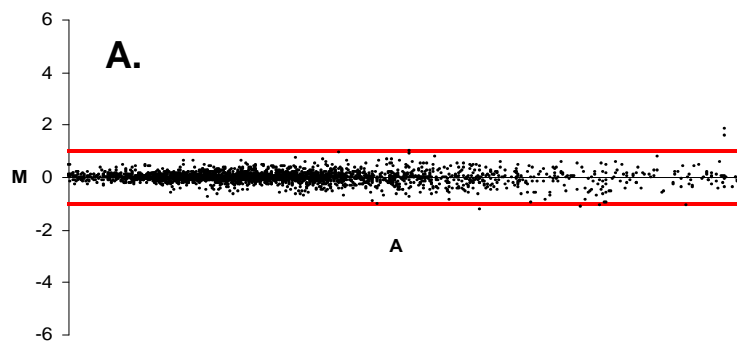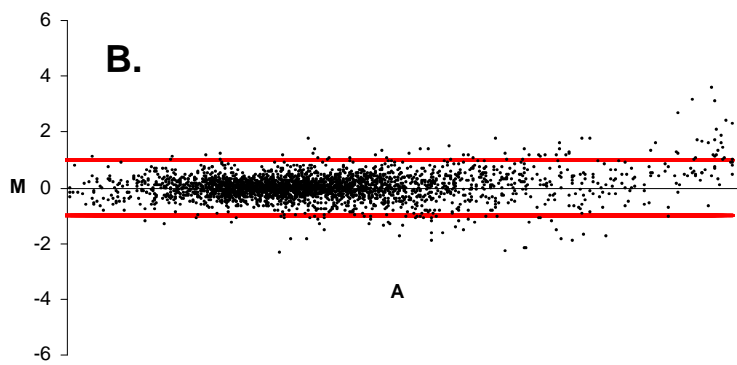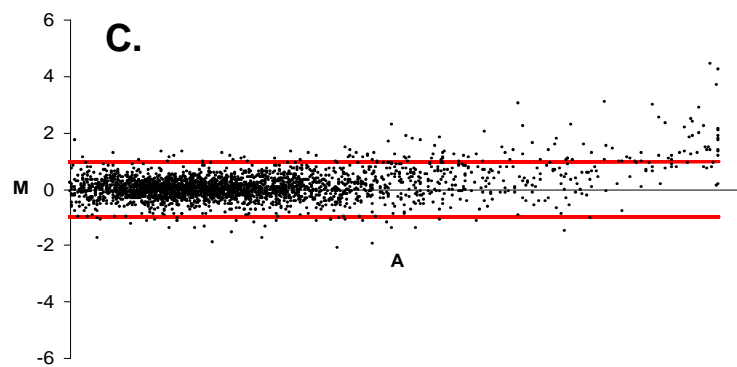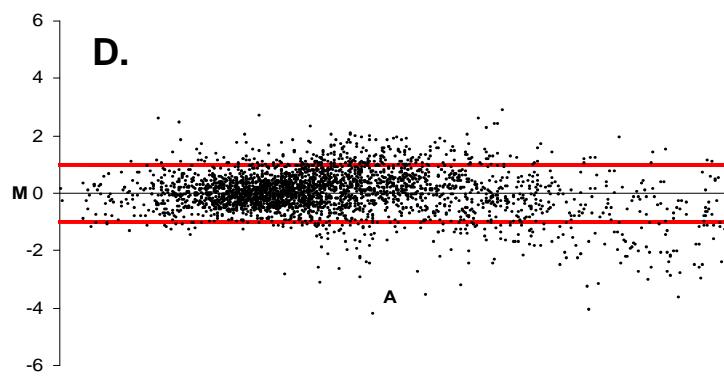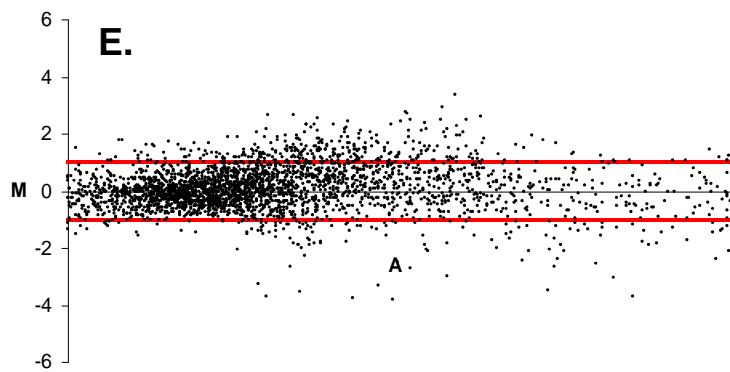

Supplement: Additional file 11 — Graphical representations of relative gene expression against fluorescence intensity from control and copper-exposed samples. Array data were normalised and filtered (as described in Methods) and the log2 of the average fold change (M) plotted against the log2 of the average mean signal intensity (A). (A)-(E) represent data from copper exposure of 0, 10, 40, 160 and 480 mg/kg, respectively. [file 1741-7007-6-25-S11.pdf]
