# Supplementary material for: 'Systems toxicology' approach identifies coordinated metabolic responses to copper in a terrestrial non-model invertebrate, the earthworm Lumbricus rubellus
Source: BMC Biol. 2008 Jun 3;6:25. doi: 10.1186/1741-7007-6-25 (PMC2424032; doi:10.1186/1741-7007-6-25)
Supplement: Additional file 2 — Chemical shift regions for integrals of lipid extract spectral data ('missing' int01 was for internal standard TMS). [file 1741-7007-6-25-S2.doc]

Additional file 2 – Chemical shift regions for integrals of lipid extract spectral data (‘missing’ int01 was for internal standard TMS)

|  | Start (ppm) | End (ppm) |
| --- | --- | --- |
| int02 | 0.567 | 0.583 |
| int03 | 0.630 | 0.658 |
| int04 | 0.666 | 0.687 |
| int05 | 0.687 | 0.704 |
| int06 | 0.764 | 0.786 |
| int07 | 0.786 | 0.806 |
| int08 | 0.806 | 0.818 |
| int09 | 0.819 | 0.845 |
| int10 | 0.845 | 0.861 |
| int11 | 0.861 | 0.875 |
| int12 | 0.875 | 0.902 |
| int13 | 0.905 | 0.930 |
| int14 | 0.930 | 0.948 |
| int15 | 0.954 | 0.994 |
| int16 | 0.997 | 1.024 |
| int17 | 1.032 | 1.116 |
| int18 | 1.116 | 1.176 |
| int19 | 1.186 | 1.412 |
| int20 | 1.435 | 1.456 |
| int21 | 1.456 | 1.478 |
| int22 | 1.478 | 1.542 |
| int23 | 1.543 | 1.609 |
| int24 | 1.610 | 1.650 |
| int25 | 1.650 | 1.714 |
| int26 | 1.715 | 1.740 |
| int27 | 1.791 | 1.877 |
| int28 | 1.884 | 1.892 |
| int29 | 1.903 | 1.915 |
| int30 | 1.923 | 1.937 |
| int31 | 1.942 | 1.973 |
| int32 | 1.973 | 2.034 |
| int33 | 2.034 | 2.135 |
| int34 | 2.196 | 2.220 |
| int35 | 2.223 | 2.263 |
| int36 | 2.263 | 2.327 |
| int37 | 2.327 | 2.368 |
| int38 | 2.743 | 2.879 |
| int39 | 3.335 | 3.463 |
| int40 | 3.481 | 3.552 |
| int41 | 3.553 | 3.588 |
| int42 | 3.590 | 3.621 |
| int43 | 3.635 | 3.656 |
| int44 | 3.658 | 3.672 |
| int45 | 3.674 | 3.716 |
| int46 | 3.870 | 4.029 |
| int47 | 4.113 | 4.125 |
| int48 | 4.135 | 4.159 |
| int49 | 4.160 | 4.183 |
| int50 | 4.184 | 4.223 |
| int51 | 4.251 | 4.341 |
| int52 | 4.341 | 4.427 |
| int53 | 5.096 | 5.160 |
| int54 | 5.160 | 5.192 |
| int55 | 5.192 | 5.264 |
| int56 | 5.274 | 5.489 |
| int57 | 6.217 | 6.258 |
| int58 | 6.482 | 6.523 |
